# Extending the R number by applying hyperparameters of Log Gaussian Cox process models in an epidemiological context to provide insights into COVID-19 positivity in the City of Edinburgh and in students residing at Edinburgh University

**DOI:** 10.1371/journal.pone.0291348

**Published:** 2023-11-21

**Authors:** Megan Ruth Laxton, Glenna Nightingale, Finn Lindgren, Arjuna Sivakumaran, Richard Othieno

**Affiliations:** 1 School of Mathematics & Statistics, University of Glasgow, Glasgow, United Kingdom; 2 School of Health in Social Science, University of Edinburgh, Edinburgh, United Kingdom; 3 School of Mathematics and Statistics, University of Edinburgh, Edinburgh, United Kingdom; 4 NHS Lothian, Department of Public Health and Health Policy, Scotland, United Kingdom; University of Zurich, SWITZERLAND

## Abstract

The impact of the COVID-19 pandemic on University students has been a topic of fiery debate and of public health research. This study demonstrates the use of a combination of spatiotemporal epidemiological models to describe the trends in COVID-19 positive cases on spatial, temporal and spatiotemporal scales. In addition, this study proposes new epidemiological metrics to describe the connectivity between observed positivity; an analogous metric to the R number in conventional epidemiology. The proposed indices, *R*_*spatial*_, *R*_*spatiotemporal*_ and *R*_*scaling*_ will aim to improve the characterisation of the spread of infectious disease beyond that of the COVID-19 framework and as a result inform relevant public health policy. Apart from demonstrating the application of the novel epidemiological indices, the key findings in this study are: firstly, there were some Intermediate Zones in Edinburgh with noticeably high levels of COVID-19 positivity, and that the first outbreak during the study period was observed in Dalry and Fountainbridge. Secondly, the estimation of the distance over which the COVID-19 counts at the halls of residence are spatially correlated (or related to each other) was found to be 0.19km (0.13km to 0.27km) and is denoted by the index, *R*_*spatial*_. This estimate is useful for public health policy in this setting, especially with contact tracing. Thirdly, the study indicates that the association between the surrounding community level of COVID-19 positivity (Intermediate Zones in Edinburgh) and that of the University of Edinburgh’s halls of residence was not statistically significant. Fourthly, this study reveals that relatively high levels of COVID-19 positivity were observed for halls for which higher COVID-19 fines were issued (Spearman’s correlation coefficient = 0.34), and separately, for halls which were non-ensuite relatively to those which were not (Spearman’s correlation coefficient = 0.16). Finally, Intermediate Zones with the highest positivity were associated with student residences that experienced relatively high COVID-19 positivity (Spearman’s correlation coefficient = 0.27).

## Introduction

The observation of infectious disease incidence in space is frequently accompanied by spatial correlation. The analyses of count data such as the counts of disease per measurement station can provide an indication of the central tendency and spread of the observed counts and the quantiles associated with the data. Choropleths provide an indication of the spatial context by illustrating the spatial proximity of the observed counts.

In this study we present three novel epidemiological indices, R spatial, R spatiotemporal, and R scaling. These are all based on hyperparameters in a Log Gaussian Cox process model. This section includes a discussion of Log Gaussian Cox process models and the importance of each index. In later sections we will show how the proposed indices can be applied to the context of COVID-19 positivity in University halls of residence.

### Literature review

#### Spatiotemporal analytical approaches

Various approaches to investigating spatiotemporal epidemiological trends exist [1–3]. Overall, the approaches highlighted by the aforementioned authors for spatial modeling include spatial interpolation, spatial statistical modeling and spatiotemporal statistical modeling. The most common methods are cast in a Bayesian framework and are namely, spatial interpolation methods and GLMM spatiotemporal models.

Spatial interpolation [4] involves estimating the value of a variable at geographic aggregations based on observed values. This method employs smoothing techniques to address the observation of extreme values in some of the geographic aggregations. Cast in a Bayesian context, this method allows for inputting expert, prior information.

The GLMM spatiotemporal models [5] observed were all Poisson based and contained the facility for the inclusion of random effects.

The BYM models [6], also Poisson based, are commonly used to estimate disease incidence/mortality in conjunction with disease/mortality mapping is an example of the abovementioned class of models. These models incorporate random effects as well, but unlike the Log Gaussian Cox Processes which we propose, these models are only specific to aggregated spatial data and not point pattern based data.

The proposed models which are Log Gaussian Cox Processes (or models) are already existing spatiotemporal models. These are commonly used in ecology [7], geography [8], and climate related research [9]. The uniqueness of our approach is to apply all of the model parameters to the epidemiological setting. So, in addition to estimating the effect of a given covariate (the traditional approach) on disease incidence—we take this further and apply the hyperparameters of the model to the epidemiological context. Typically, in spatial epidemiology aggregated data is used and incidence rates are estimated (estimates) using the BYM models. Our approach is to use both point and aggregate data—hence our marked point patterns as the data.

#### Extending the R number

In conventional epidemiology the reproduction number is described as [10] is: “the expected number of new infections produced by a single (typical/average) infectious individual, when introduced into a totally susceptible population”. Note that the basic reproduction number *R*_0_, is applicable to situations where there is no immunity [11]. The importance of the *R*_0_ in public health was highlighted during the COVID-19 pandemic. Globally, governments and public health decision makers used *R*_0_ as part of the body of evidence to inform COVID-19 public health policy. In particular *R*_0_ will help policymakers decide whether a given community will be impacted by the disease at hand [12] and also which fraction of the community is estimated to be vaccinated as a protective measure. When *R*_0_ is greater than 1, the implication is that the disease will spread. There are various methods for the estimation of *R*_0_ [10, 13] and these are underpinned by ordinary differential equations [14]. The SIR modeling approach for example, is one such method, and involves the division of the population at hand (N), into three compartments; S (those individuals who are susceptible to the disease), I(those who are infectious), and R (those who have recovered or have been removed). Various statistical software, such as the R can facilitate the computation necessary. Our proposed indices will act as a supplement to the basic reproduction number, *R*_0_ because they will provide spatial insights into the spread of the disease at hand. *R*_0_ gives an indication of the spread of the disease between individuals (under the conditions previously discussed), whilst our proposed indices give an indication of the distance over which cases of infections are correlated. Together these metrics will help the public health decision maker in terms of assessing the risk of a community to infections, and assessing the distance over which cases can be linked with each other. The latter objective might be useful in establishing localized lockdowns. Similar to *R*_0_, our new indices can be affixed with a subscript 0 when the situation is such that there is no immunity in the community. We propose the use of credible intervals when these new indices are used/published. **R spatial**—an indication of the distance over which cases are correlated with each other and serve as an estimation of the extent of the spread of the virus within halls of residence (a spatial R number) **R spatiotemporal**- indicates the correlation in the spatial distribution of COVID-19 positivity as the timeline progresses. The positive estimate of this index indicates that as the months progress from September towards December the COVID19 positivity will be correlated to the previous month. **R scaling**—defines the strength and direction of the interaction between density of university halls of residence and residence COVID-19 levels. For the chosen model, R scaling is estimated to have a posterior mean value of 1.91, with 95% CI between 0.61 and 3.28. This suggests that areas with higher densities of student halls are likely to have higher COVID-19 levels than areas with lower densities of halls. This index can be useful in comparative studies where the numerical value of R scaling for different universities for example, can be compared and inform related policy. Overall, this index provides an indication of the trend (higher vs. lower) of disease cases relative to the density of spatial units (university halls or residential buildings etc).

### Study setting

In terms of COVID-19 incidence at university halls of residence, research so far has indicated that the influx of first year students might be an important factor in terms of the spread of the disease, most likely due to living in halls of residences [15]. It should be mentioned however, that the University community from which this research has its basis is the University of Bristol. In the Bristol study, the use of stochastic transmission models [15] has suggested that first year university students were the main drivers of the transmission of COVID-19. Additionally, these researchers suggest that the most effective intervention for reducing transmission is to limit or reduce face to face teaching. Interestingly, using SEIR models, researchers [16] have discussed the successful control of COVID-19 transmission in an American university, Boston University, despite an accelerating in the rate of transmission in the surrounding community, the City of Boston.

The above-mentioned studies highlight the importance of examining the association of student influx on COVID-19 spread. They do not however, infer that there is a link between the influx of first year students on COVID-19 spread for all or most universities in general. The current study will investigate the situation for one University, the University of Edinburgh where 17184 students were enrolled in the autumn of 2020.

An analysis of the observed counts per measurement station and simultaneously, that of the unobserved spatial correlation, represented using a Gaussian Random Field, necessitates a modeling approach which incorporates both components. This study seeks to model the observed counts of COVID-19 positivity in students living in close proximity in University of Edinburgh halls of residence whilst accounting for the spatial correlation of the levels of positivity per hall and for the spatial correlation of the location of the hall. This approach is potentially valuable to public health policy and University health and safety policy since the model provides an estimate of the spatial range at which the levels of positivity per hall are correlated (or connected) as well as the spatial correlation of the hall locations. This information can be used to inform decisions on implementing localized lockdowns, for example.

The models will also investigate whether the spatial correlation of COVID-19 positivity varies by residence category, that is, ensuite or non-ensuite categories. The number of rooms per hall is used as an offset in the models to account for the density of students. A secondary goal for this study is to examine the association between the positivity levels at halls of residence and that of the surrounding community. In the case of Edinburgh University, the surrounding community would be represented by the Intermediate Zones where each hall/residence is represented by. Note that Intermediate Zones are stable geographies which fall between Data Zones and Local Authority geographies.

The proximity of services such as supermarkets, shopping centres, gyms, and restaurants to halls of residence where students have recently taken up residence may serve as a pathway of epidemiological interaction (epiaction) between halls of residence and the community. The extent of the epiaction would depend on the zone of influence [17] of the service, the activity (social interaction and shared amenities) within the residences and the positivity levels in neighbouring Intermediate Zones.

Importantly epiaction can be symmetric or asymmetric. The use of multiple analytical tools in tandem is the ideal approach to deciphering the direction of epiaction in this scenario. The inclusion of temporal variables such as intervention measures would add to the usefulness of this model. Overall, universities bring together students that come from a variety of settings locally, nationally and internationally—this makes them a potential vector for transmission of infection to and from the places of their origin. The dynamics associated with student teaching, studying and dining arrangements are also potentially important factors that may facilitate easy transmission of infection. Understanding and risk assessing these dynamics are important in the control of COVID-19 in these institutions of higher learning.

## Research questions

The study timeframe is September 2020 to December 2020 and the study research questions are outlined below: (i) Are there any spatial patterns of COVID-19 positivity in the halls of Edinburgh University and the surrounding communities? (ii) Are there any differences in living in ensuite/non-ensuite on student positivity levels (Edinburgh University), and separately, in halls which had relatively higher/lower fines issued? (iii) What are the temporal positivity trends (in the presence of interventions such as mandatory face covering use, limitations in size of gatherings, and closure of hospitality venues) in the surrounding communities? (iv) What is the relationship between the COVID-19 positivity at halls of Edinburgh University and the community, and (v) What are the public health implications of the research findings?

## In the news

Thousands of students returned in person to further education in the UK /Scotland in September 2020. In Edinburgh, and the rest of Scotland, COVID-19 testing of student returners was limited to PCR testing of symptomatic cases only. Other UK universities such as Cambridge University introduced asymptomatic pooled testing of all students from autumn 2020 [18]. In this scenario, individuals tested do not display COVID-19 symptoms. Note that COVID-19 can be transmitted from infected individuals who are themselves asymptomatic.

In Scotland, outbreaks at universities were reported soon after their reopening at the start of the 2020/21 academic year, for example in Glasgow [19], Napier University [20], and in Edinburgh. In Sept 2020, large COVID-19 outbreaks [21] were reported at the majority of universities and further education institutions (The BBC, September 2020). These outbreaks were managed by Incident Management Teams (IMT) set up and chaired by consultants in Public Health from the local Health Board working jointly with key staff from the local universities.

University COVID-19 outbreaks and policies were, amongst other things, a popular topic of discussion during the autumn of 2020 [22, 23]. There were also expressions of concern regarding the level of occupancy of university halls with one newspaper (The Guardian, October 2020) publishing the headline “UK university student halls too full to be safe, experts warn”. In this report, an individual is quoted as saying “reopening a university is like dumping a cruise ship in the middle of town and letting passengers off”.

The independent UK Scientific Advisory Group for Emergencies (SAGE) [24] group advised that the virus was transmitted within residential halls and via face-to-face teaching. This group recommended online instruction. Issues arose regarding students returning to their homes for the holidays, with some advising that they should remain at the university halls to minimise onward transmission into their local communities [25]. In terms of students needing to isolate, policies were set up to allow students to isolate at their homes [25, 26]. The government reached an agreement to set up policies (at the end of the academic term) to allow students who were required to isolate due to a positive test, to return to their local residence and isolate in their homes instead of remaining in halls of residence. Some universities also viewed the challenges presented by the pandemic as an opportunity to rethink the future of higher education including methods for delivery of teaching [27, 28]. In terms of students returning to universities [29], various modes of testing were used; for example, mass testing in St. Andrews [30]. At the University of Edinburgh, lateral flow testing of students began in November 2020. This followed the Scottish Government’s “test before you travel” policy on November 17th, 2020 (personal communication, University of Edinburgh). In total 28511 tests were conducted between 30th November 2020 and 30th June 2021.

In terms of seeding of infections in Scotland as a whole, some researchers indicated that new infections in Scotland during the second wave was linked to a variant discovered in Spain [31]. The UK’s travel policy in the summer of 2020 was described as being flawed as ‘the virus moves when people move’ [31]. Studies suggest that traveling outside of the UK led to reseeding of infections [32, 33]. Genomic epidemiology reveals multiple introductions of SARS-CoV-2 from mainland Europe into Scotland [32].

Finally, some described the possibility of outbreaks at University residences as an “Accident waiting to happen” due to the report that many Scottish universities did not reduce capacity [23] of student numbers in their halls of residence in order to increase social distancing.

Key local control measures are outlined in Table 1 in the Appendix.

**Table 1:** Key local control measures for outbreaks for halls of residences in Edinburgh.

Control Measures
a. Clear advice was provided on the management and self-isolation of anyone who tests positive for COVID-19, associated contact tracing of their close contacts, and outbreak response led by Health Protection Teams +/- with IMTs as needed
b. The majority of teaching of large groups was done online
c. In person teaching was done for only some subjects were done such as medical and nursing students
d. Early identification of cases by PCR testing facilitated by the university
e. Isolation of confirmed cases with support from the university
f. All students were requested to download the Protect Scotland app
g. Identification of close and social contacts with advice to isolate with support from the university
h. Communications on following ‘FACTS’, what to do to limit transmission, what to do if you have symptoms of COVID-19 and advising on limiting social gatherings to limit spread
i. Advice on social gatherings/parties
j. Advice letters on student behavior issued from universities
k. Universities increased the staff presence to enforce rules and give welfare help and advice and they will ask private providers of student accommodation to do the same
l. Joint working with local police to discourage large gatherings and parties
m. At Edinburgh universities sanctions were applied to students who persistently refused to follow rules (unpublished IMT notes from NHS Lothian Health Board)
n. Police Scotland were asked to keep an eye on student behavior off campus (relating to parties and large gatherings) and a strict “yellow card/red card” system was brought in to deal with breaches that put students and others at risk
o. The principals of all of Scotland’s universities and the Scottish government agreed on some tough new rules for students This included limitations on socialization where students were asked to refrain from socializing with anyone outside their household That meant no parties, no visits to the pub, restaurants, or any other hospitality venues on the aforementioned weekend
p. Advice to students during the last week of September 2020 to avoid socializing in pubs, cafes, and restaurants, and to socialize instead within their households. This was advised for one weekend
q. Amidst the outbreaks and interventions, many news outlets reflected various sentiments about the control of the transmission at halls of residences
r. Plans were made for testing students before departure from university and for testing students on return to university with a staggered arrival
s. In December 2020, Scottish universities set up COVID-19 LFD testing facilities for students returning to their families for the Christmas holidays Results were texted to students
t. By January 2021 Post Christmas, students asked not to return to Halls until Feb/March 2021 Remote teaching was done during this time. Note that this is in relation to the Alpha variant
u. Finally, in December 2020, it was decided that students starting clinical/professional placements would be allowed to return to the University in January 2021, following the Christmas holidays in a staggered fashion over a period of at least six weeks

Tables 2 and 3 show the number of rooms at the University of Edinburgh halls of residence during the study period, and enrolment per month during the study timeframe respectively.

**Table 2:** Number of rooms at University of Edinburgh halls of residence.

Type of room	Number of rooms
Ensuite	5139 with 3675 being cluster flats or studios
Non-ensuite	4702, 3776 of which are in cluster flats

**Table 3:** Student enrolment by month in the study timeframe.

Month	Enrolment
September 2020	16788
October 2020	286
November 2020	48
December 2020	62

## Method

The proposed methods include data visualization (choropleths, heatmaps) and point process models, which are ideal for point pattern data where locations of observed events are provided. The spatial distribution of residences is modelled using a Log Gaussian Cox Process (LGCP) model, which is doubly stochastic by nature -comprising of a Poisson point process, and a spatial random field [34, 35]. The proposed suite of 8 nested models are marked point process models, extending the LGCP model to jointly model the locations of residences (the point pattern) alongside the count of COVID-19 positivity (a property measured at the residence locations, called a ‘mark’) with two dependent likelihoods. Apart from the parameters associated with the covariates in the models, the parameters of the random fields representing residence distribution and COVID-19 count distribution are important in that they provide an estimate of the spatial range over which the observed counts are correlated and the effect of residence clusters on COVID-19 positivity. We propose to demonstrate how the parameters associated with the random fields can be useful epidemiological indices. These indices, *R*_*spatial*_, *R*_*spatiotemporal*_, and *R*_*scaling*_ represent the modeled posterior estimate of the range of the spatial field, temporal correlation and scaling parameter respectively.

Finally, ethics approval was given by the University of Edinburgh’s Ethics Committee (in Nursing Studies) on 12th November 2021 with an ID: NUST011s.

### Data

The data for this study has been provided by the University of Edinburgh’s Accommodation, and Health and Safety divisions. Data has been provided on the number of students who tested positive by PCR for COVID-19 positivity per university owned residences over 8 months starting in September 2020. Data for 51 residences was obtained. This study focuses on the months September to December 2020. Information on the halls of residence (such as ensuite status) was provided for the residences within halls. The location (latitude and longitude) of each hall of residence was obtained from Google maps. The data has been provided at an aggregated level so individuals cannot identify themselves in the analyses. Finally, data on the number of COVID-19 fines issued per hall of residence during the study period was also provided by the Univeristy. Note that these fines were issued by the University’s Accommodation, Catering and Events (ACE) team. For this study, the data is stored on a dedicated laptop and after this study the data will be destroyed. The intended output of the study includes:

- Visual illustrations of the time series of COVID-19 positivity in the City of Edinburgh and separately, in Intermediate Zones within the City of Edinburgh,
- Visual illustrations of the spatial trend of COVID-19 positivity in Intermediate Zones within the City of Edinburgh,
- Visual illustrations of the estimated spatial correlation of COVID-19 positivity in the University of Edinburgh halls of residence,
- Modeled estimates of the spatial range and standard deviation of the spatial random field underlying the data, and an
- Interpretation of the estimated spatial fields and the implications for public health policy and University health and safety policy.

### Models

Here, we aim to answer the research questions detailed earlier (in the section on Research Questions) by fitting a series of 8 nested marked point process models to data on the counts of COVID-19 positivity by university hall of residence between September 2020 and December 2020. Details of the models are provided in Table 4.

**Table 4:** The proposed suite of 8 nested models.

Model	Spatial	Temporal	Community	Ensuite	Fine	Hall Group
Model 1	✓					
Model 2	✓		✓			
Model 3	✓	✓				
Model 4	✓	✓	✓			
Model 5	✓	✓	✓	✓		
Model 6	✓	✓	✓		✓	
Model 7	✓	✓	✓			✓
Model 8	✓	✓	✓	✓		✓

#### Mark likelihood

Models 1 and 2 are purely spatial models, which follow the form:

$$\begin{array}{c} y(s)∼Poissonon(\mu (s)) \end{array}$$ (1)

Where, *y*(*s*) represents the total count of COVID-19 cases observed at a residence at location *s* (during the study period).

Models 3–8 incorporate a temporal correlation structure, and model the data in space *and* time. Models 3–8 follow the form:

$$\begin{array}{c} y(s,t)∼Poissonon(\mu (s,t)) \end{array}$$ (2)

Where, *y*(*s*, *t*) represents the total count of COVID-19 cases observed at a residence at location *s* and time *t* (during the study period). The structure of all 8 models is outlined in Table 5.

**Table 5:** Structure of the mark likelihood for the 2 spatial and 6 spatiotemporal models.

1	$log\left(\mu \left(s\right)\right)=\alpha_{1}+\kappa \psi \left(s\right)+\omega \left(s\right)$
2	$log\left(\mu \left(s\right)\right)=\alpha_{1}+\beta_{i}x_{i}\left(s\right)+\kappa \psi \left(s\right)+\omega \left(s\right)$
3	$log\left(\mu \left(s,t\right)\right)=\alpha_{1}+\kappa \psi \left(s\right)+\omega \left(s,t\right)$
4	$log\left(\mu \left(s,t\right)\right)=\alpha_{1}+\beta_{i}x_{i}\left(s,t\right)+\kappa \psi \left(s\right)+\omega \left(s,t\right)$
5	$log\left(\mu \left(s,t\right)\right)=\alpha_{1}+\beta_{i}x_{i}\left(s,t\right)+\beta_{j}x_{j}\left(s\right)+\kappa \psi \left(s\right)+\omega \left(s,t\right)$
6	$log\left(\mu \left(s,t\right)\right)=\alpha_{1}+\beta_{i}x_{i}\left(s,t\right)+\beta_{l}x_{l}\left(s,t\right)+\kappa \psi \left(s\right)+\omega \left(s,t\right)$
7	$log\left(\mu \left(s,t\right)\right)=\alpha_{1}+\beta_{i}x_{i}\left(s,t\right)+\beta_{m}x_{m}\left(s,t\right)+\kappa \psi \left(s\right)+\omega \left(s,t\right)$
8	$log\left(\mu \left(s,t\right)\right)=\alpha_{1}+\beta_{i}x_{i}\left(s,t\right)+\beta_{j}x_{j}\left(s\right)+\beta_{m}x_{m}\left(s,t\right)+\kappa \psi \left(s\right)+\omega \left(s,t\right)$

In all 8 models (Table 5), *α*_1_ represents an intercept term; *ψ* is a spatially structured random field that also appears in the linear predictor for the spatial pattern; and *κ* is a scaling parameter. In Models 1–2, *ω* denotes a spatially structured random field that is unique to the counts of COVID-19 cases per residence (i.e. the ‘marks’ of the point pattern). Both random fields (*ψ* and *ω*) follow a Matérn covariance structure with spatial range parameter *ρ* and standard deviation *σ*. In Models 3–8, the *ω* field is spatiotemporally structured, incorporating an AR1 process with correlation parameter *ρ*_*t*_.

In the section on Novel epidemiological Indices, we introduce 3 novel epidemiological indices, derived from the parameters of random effects in the models. We refer to the mean posterior estimate of the spatial range (*ρ*) of the mark field (*ω*(*s*) and *ω*(*s*, *t*)) as *R*_*spatial*_; the mean posterior estimate of the temporal correlation parameter (*ρ*_*t*_) from the AR1 process of the spatiotemporally structured random field (*ω*(*s*, *t*)) as *R*_*spatiotemporal*_; and the mean posterior estimate of the scaling parameter (*κ*) as *R*_*scaling*_.

In Model 2, *x*_*i*_(*s*) represents the value at location *s* of the covariate quantifying the number of COVID-19 cases in the community per Intermediate Zone. A spatiotemporal version of this covariate is included in Models 4–8.

In Models 5 and 8, *x*_*j*_(*s*) represents the value at location *s* of the covariate quantifying the proportion of total rooms with ensuite facilities per residence.

In Model 6, *x*_*l*_(*s*, *t*) represents the value at location *s* and time *t* of the covariate quantifying the number of fines received by each residence for breach of COVID-19 regulations.

In Models 7 and 8, *x*_*m*_(*s*, *t*) represents the value at location *s* and time *t* of the binomial covariate indicating whether or not the residence is part of one of the largest halls (unnamed) on campus.

The regression coefficients (*β*_*i*_, *β*_*l*_, *β*_*j*_, and *β*_*m*_) of covariates are estimated in the models.

#### Point likelihood

The intensity of the point pattern for all models is modelled as:

$$\begin{array}{c} \lambda \left(s\right)=exp(\alpha_{0}+\psi \left(s\right)) \end{array}$$ (3)

Here *α*_0_ is another intercept, and *ψ*(*s*) is the same spatially structured Gaussian random field as found in the model structures above.

### Prior specifications

In this study, PC-priors are specified to inform the Matérn covariance structure of the Gaussian Random Fields *ψ* and *ω*. PC-priors penalise complexity away from a simpler base model, so ensure that the effects of complex model components (such as Gaussian Random Fields) are only included when this is necessitated by the data [36]. In the case of the Matérn field, a PC-prior is specified on the joint density of the spatial range (*ρ*) and marginal standard deviation (*σ*^2^). The spatial range gives an indication of the spatial span of the covariance structure, and the standard deviation provides the degree of spatial variability. The hyper-parameters (*R* and *S*) for this prior are set when using inlabru, the R software package used for this analysis [37, 38]. This is done indirectly by specifying the lower tail quantile and probability for the range, and the upper tail quantile and probability for the standard deviation.

In the R code for the analysis, the user must specify:

prior.range = c(*ρ*_0_,*p*_*ρ*_)

Where *ρ*_0_ corresponds to the lower tail quantile for the range, and *p*_*ρ*_ corresponds to the probability that the actual range value (*ρ*) is less than *ρ*_0_. This can be written as:

$$\begin{array}{c} P\left(\rho <\rho_{0}\right)=p_{\rho } \end{array}$$ (4)

Similarly, for the standard deviation, the user must specify:

prior.sigma = c(*σ*_0_,*p*_*σ*_)

Where *σ*_0_ corresponds to the upper tail quantile for the standard deviation, and *p*_*σ*_ corresponds to the probability that the actual standard deviation (*σ*) is greater than *σ*_0_. This can be written as:

$$\begin{array}{c} P\left(\sigma >\sigma_{0}\right)=p_{\sigma } \end{array}$$ (5)

This means that users are able to inform both the locations of the tails *and* their certainty about this. For the models fitted in this paper, the priors on the parameters for both random fields (*ψ* and *ω*) were *P*(*ρ* < 0.2) = 0.01 and *P*(*σ* > 1) = 0.01, where the lower tail location for the range parameter is given in units of km.

For the Matérn field, PC-priors penalise complexity away from the base model in which the random field is spatially constant (*ρ* = ∞) and has zero variance (*σ* = 0). This means the effects of a field with the user-specified priors on the parameters above are only included if this is supported by the data.

## Results

### Temporal trends

**Fig 1:**
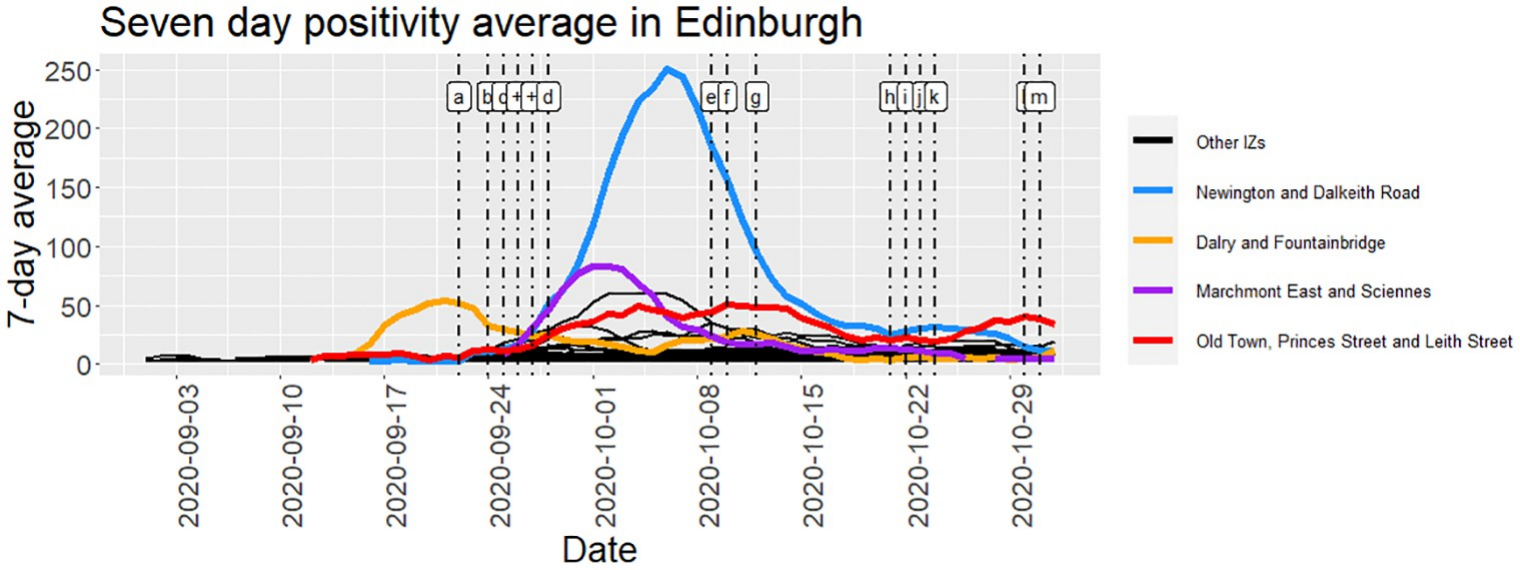
Time series of positivity (COVID-19) in Edinburgh by Intermediate Zone with key policies indicated. The policies are indexed as follows: a. Ban on visiting other households; b. Uni students advised to socialise amongst acc.grp.; c. Police patrols to enforce rules; d. Student guidance issued for visiting parents; e. More student guidance on visiting parents; f. Bars/restaurnts/pubs close early till Oct 25; g. Restrictions on Glasgow prison; h. Care home rules relaxed; i. Restrictions on hospitality businesses extended; j. Digital Christmas advised; k. Five tier Scottish COVID-19 system announced; l. Halloween discouraged; m. Face coverings advised for senior pupils and teachers.

**Fig 2:**
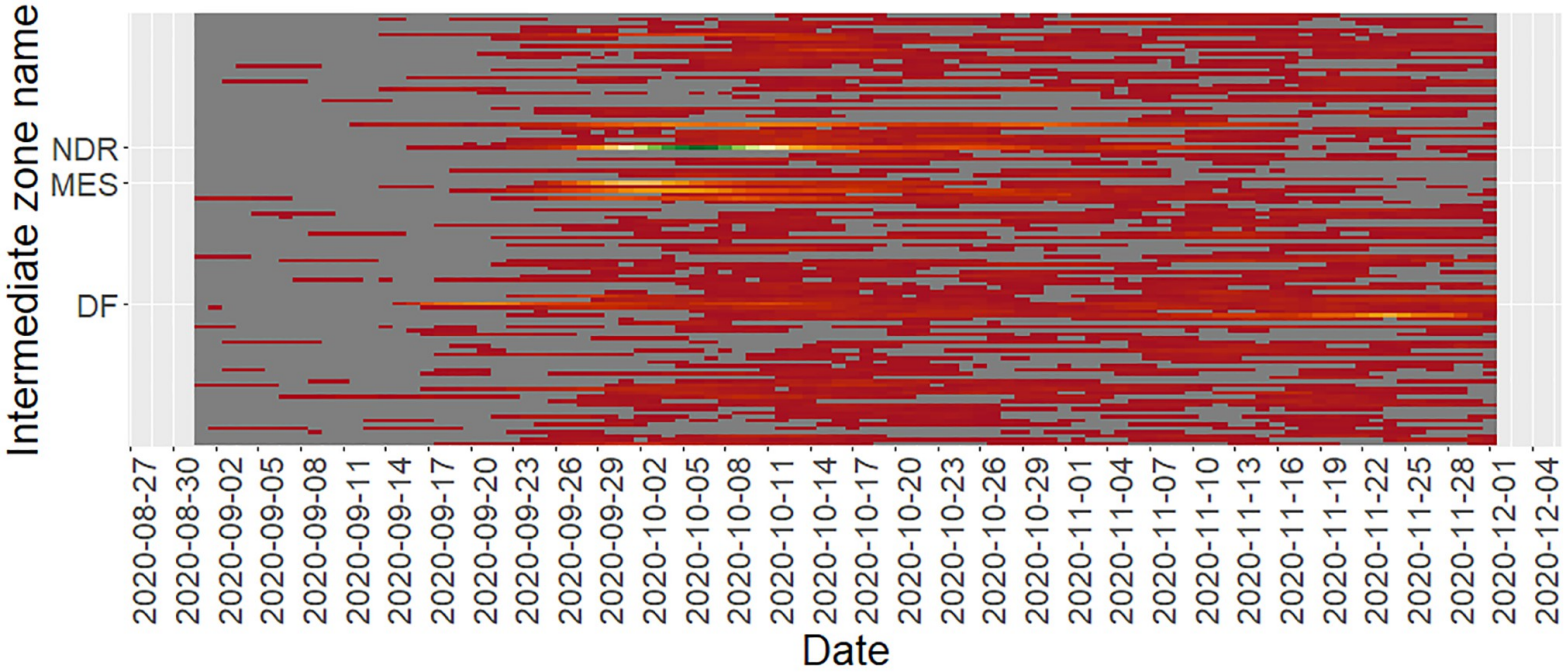
Heatmap with date on the x-axis and Intermediate Zones on the y-axis. The symbols NDR, MES, and DF on the y-axis represent the Intermediate Zones, Newington, and Dalkeith Road, Marchmont East and Sciennes, and Dalry and Fountainbridge. Note that Zones with NA as value were filtered off.

Here we provide an indication of the trend of COVID-19 positivity in the City of Edinburgh between September 1st 2020 to October 31st 2021. This is provided against the backdrop of the chronology of COVID-19 public health interventions in Scotland (See Figs 1 and 2) with Intermediate Zones of relatively higher positivity highlighted. From Fig 1 we note that public health policies in Scotland included a 77-day lockdown (restricting social and spatial interaction of residents) and later the use of face coverings and were mostly, layered interventions [15]. In addition, university students were encouraged (by email) to avoid socializing in pubs and restaurants during the weekend of September 26th, 2020. This timeframe is denoted as “++” in Fig 2. Note that the university was closed during the spring term.

Fig 1 shows the evolution of COVID-19 positivity in Edinburgh against the backdrop of public health policy. In the study timeframe, three prominent peaks are observed in the figure. The first peak in late September 2020 is observed at Dalry and Fountainbridge, whilst the second (relatively larger peak) is observed at Marchmont East and Sciennes. The third peak (highest) is at Newington and Dalkeith in early October 2020. In addition to observing distinct peaks, it was observed that the trend for the Intermediate Zone, Old Town, Princes Street and Leith Street, plateaued for approximately 21 days (longer than the abovementioned peaks) before declining.

The heatmap in Fig 2 provides an indication of the temporal trend between the study period with separate rows for each Intermediate Zone. The legend for this heatmap is suppressed to avoid any statistical disclosures. The highest count range is green, the medium range is yellow, and the lowest, red. From the heatmap the comparatively higher positivity is observed in 2021, whilst in 2020, a sustained “spark” is observed in October 2020 with a progression in positivity towards the end of the year. That spark is represented by the Intermediate Zone, Newington, and Dalkeith Road. Earlier sparks of shorter duration were observed as well, such as in Dalry and Fountainbridge and increasing for Marchmont East and Sciennes.

### Spatial trends

**Fig 3:**
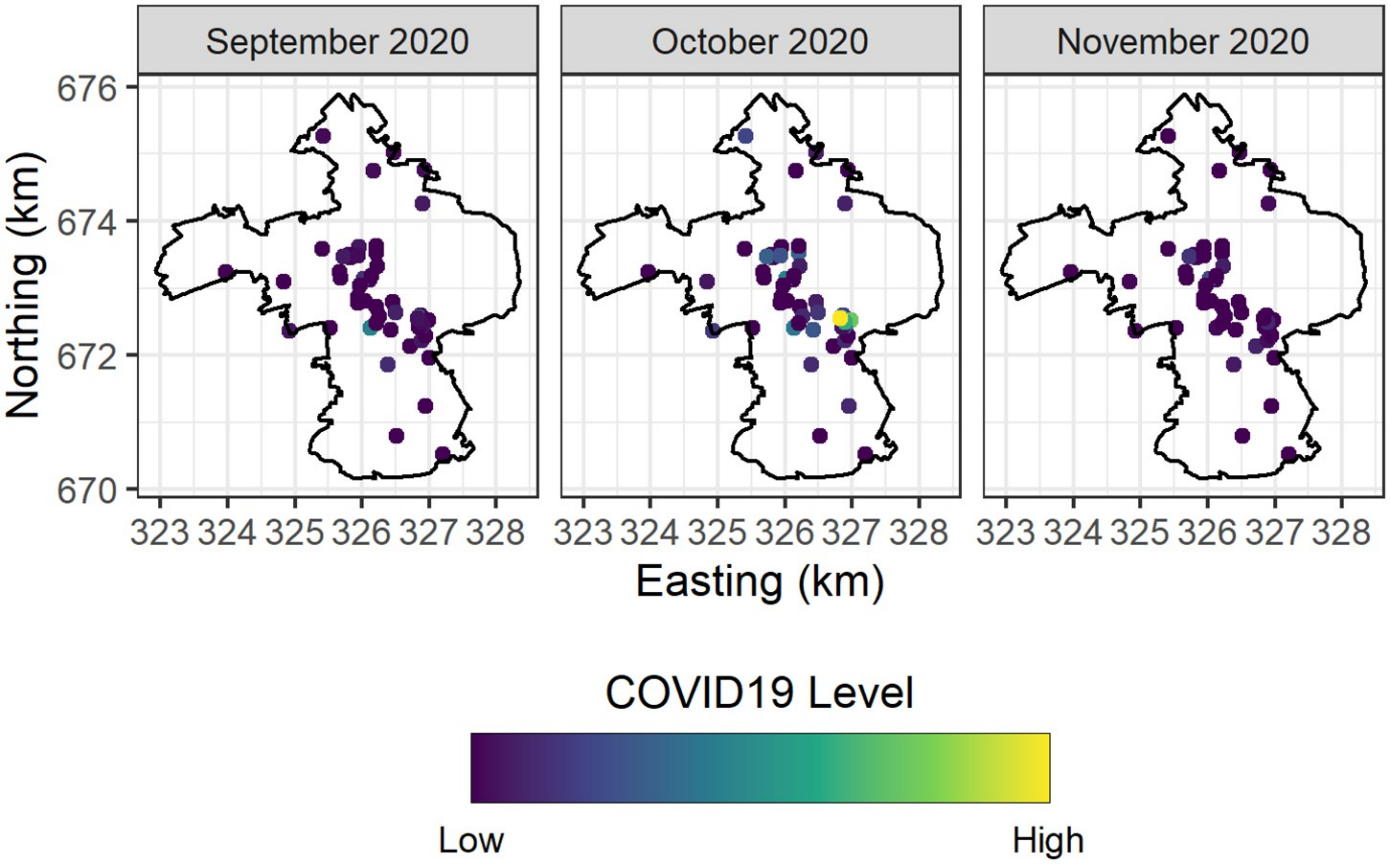
Spatial trends in COVID-19 positivity across University of Edinburgh student residences.

**Fig 4:**
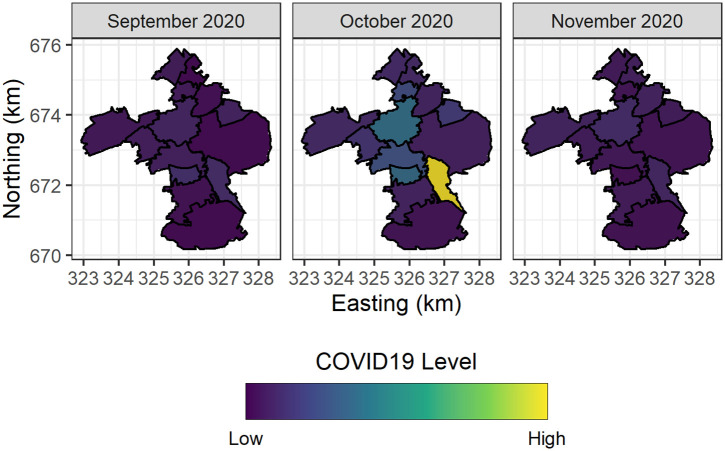
Spatial trends in COVID-19 positivity across the surrounding communities of university owned student residences.

Figs 3 and 4 depict the spatiotemporal trends of COVID-19 positivity (counts) in 51 University of Edinburgh owned student residences, and the spatiotemporal trends in COVID-19 positivity (7-day average) across Intermediate Zones in the City of Edinburgh respectively.

In Figs 3 and 4 it is clear that there were flare-ups in COVID-19 positivity in October 2020 in both the Intermediate Zones in the City of Edinburgh and within the University of Edinburgh owned student residences. Importantly we note that the relatively higher positivity levels are observed to be spatially clustered in similar locations. In particular, the Intermediate Zone with the highest level of positivity in October 2020 is Newington, and Dalkeith Road—this is the immediate spatial neighbourhood (community) of the University of Edinburgh student residences which experienced the highest levels of positivity in the aforementioned month.

### Modeling

#### Model discrimination

We considered a suite of 8 nested marked point process models. Based on the model diagnostics and principles of parsimony, the model with the lowest Widely applicable information criterion (WAIC) [39] value is Model 4, indicating that this model provides the best balance between goodness of fit and model simplicity (Table 6). Model 8 has a similar WAIC (higher than model 4 by 7 WAIC points). Based on Spiegelhalter’s advice on DIC [40], the models 4 and 8 can be considered different from each other. Model 4 contains a component to account for spatiotemporal correlation structures in the data, and a covariate for the community COVID-19 level. It is important to note here that the meaning of scores such as WAIC in this modelling context remains largely unexplored, and that modelling aims and biological interpretability should also be considered in the model selection process.

**Table 6:** Posterior mean and 95% credible intervals for: scaling parameter/*R*_*scaling*_ (*κ*); spatial range (*R*_*spatial*_) and SD of the random fields (*ω* and *ψ*); temporal correlation/(*R*_*spatiotemporal*_) of the AR1 process (*ρ*_*t*_); and regression coefficients(*β*_*i*_,*β*_*j*_,*β*_*l*_,*β*_*m*_,) of covariates: number of COVID-19 cases in the community per Intermediate Zone (*x*_*i*_); proportion of total rooms with ensuite facilities per residence (*x*_*j*_); number of fines received by each residence for breach of COVID-19 regulations (*x*_*l*_); and whether or not the residence is part of one of the largest halls (unnamed) on campus (*x*_*m*_). Model run times (seconds), Watanabe–Akaike information criterion (WAIC) scores, and log conditional predictive ordinate (log-CPO) scores are also given. All values given are rounded to 2 decimal places.

Parameter	Model 1	Model 2	Model 3	Model 4	Model 5	Model 6	Model 7	Model 8
*κ*	-2.05	-2.3	1.75	1.91	1.89	4.09	1.37	1.34
	[-3.53,-0.67]	[-3.8,-0.9]	[0.44,3.14]	[0.61,3.28]	[0.59,3.27]	[2.91,5.37]	[-0.03,2.82]	[-0.07,2.81]
*ω* Range	0.26	0.24	0.18	0.19	0.18	0.49	0.19	0.19
	[0.18,0.37]	[0.17,0.33]	[0.14,0.23]	[0.13,0.27]	[0.12,0.24]	[0.35,0.66]	[0.13,0.26]	[0.13,0.26]
*ω* SD	2.28	2.03	2.56	2.53	2.53	1.33	2.64	2.64
	[1.71,3.05]	[1.49,2.65]	[2,3.22]	[1.87,3.33]	[2.1,3.03]	[0.98,1.8]	[2.12,3.26]	[2.18,3.19]
*ψ* Range	0.46	0.45	0.46	0.46	0.48	0.42	0.47	0.47
	[0.36,0.59]	[0.36,0.55]	[0.37,0.57]	[0.38,0.55]	[0.37,0.62]	[0.35,0.5]	[0.37,0.59]	[0.36,0.59]
*ψ* SD	2.89	2.86	2.9	2.92	2.91	3.08	2.89	2.88
	[2.36,3.51]	[2.25,3.54]	[2.31,3.6]	[2.46,3.48]	[2.34,3.64]	[2.46,3.82]	[2.25,3.69]	[2.22,3.71]
*ρ*_*t*_	NA	NA	0.49	0.48	0.49	-0.37	0.54	0.55
			[0.36,0.6]	[0.37,0.59]	[0.4,0.58]	[-0.59,-0.17]	[0.4,0.66]	[0.46,0.63]
*β*_*i*_	NA	4.59	NA	-6.11	-5.97	-13.22	-4.45	-4.4
		[-0.58,9.6]		[-50.09,37.86]	[-49.95,38]	[-57.22,30.77]	[-48.44,39.53]	[-48.38,39.59]
*β*_*j*_	NA	NA	NA	NA	0.07	NA	NA	0.06
					[-0.43,0.57]			[-0.43,0.56]
*β*_*l*_	NA	NA	NA	NA	NA	-0.05	NA	NA
						[-0.09,-0.02]		
*β*_*m*_	NA	NA	NA	NA	NA	NA	-0.48	-0.43
							[-3.2,2.11]	[-3.12,2.16]
Run Time	128.8	135.52	1404.75	2347.98	1654.3	4043.06	1410.46	1362.36
WAIC	2448.43	2419.69	1807.76	1777.29	1858.74	1989.32	1828.76	1785.38
log-CPO	2.6106735^6^	2.6214256^6^	2.6569091^6^	2.662974^6^	2.657011^6^	2.7392473^6^	2.6655611^6^	2.6679214^6^

#### Covariates

In all 8 models, all of the 95% credible intervals for the covariate effects crossed 0, indicating that none of the covariates had a statistically significant effect on the response (Table 6).

The community level covariate was hypothesised to be of particular importance due to its coverage in the media. However, for the 6 spatiotemporal models considered here, the credible intervals for this covariate effect were very large, meaning that no statistically significant inferences could be made about the effect of this covariate. In the spatial model which included this covariate (Model 2), the posterior mean regression coefficient is positive, suggesting that as the COVID-19 positivity increases in the community, there is an expected increase in the COVID-19 positivity at the university halls of residence. While the 95% credible interval for *β*_*i*_ in Model 2 is smaller than that of the spatiotemporal models, it does cross 0, so this inference is not supported by statistical significance.

#### Novel epidemiological indices

We use the terms *R*_*spatial*_, *R*_*spatiotemporal*_ and *R*_*scaling*_ to represent the mean posterior estimates of the mark field (*ω*) spatial range (*ρ*), the temporal correlation (*ρ*_*t*_), and the scaling parameter (*κ*), respectively. These are the three indices that we propose in this study to describe epidemiological spread. The posterior estimates of these values from the selected model (Model 4) are *R*_*spatial*_ = 0.19 [0.13, 0.27], *R*_*spatiotemporal*_ = 0.48 [0.37, 0.59] and *R*_*scaling*_ = 1.91 [0.61, 3.28], respectively (Table 6).

*R*_*spatial*_ is given in the spatial units of the input data (here, km). Thus, we can interpret the range in connectivity in COVID-19 levels between university halls of residence in Edinburgh as an average of 0.19km, with 95% CI between 0.13km and 0.27km. The mean nearest neighbour distance between the halls of residence considered in this study is 0.23km, with the distances between halls ranging from 0.003km to 5.061km.

**Fig 5:**
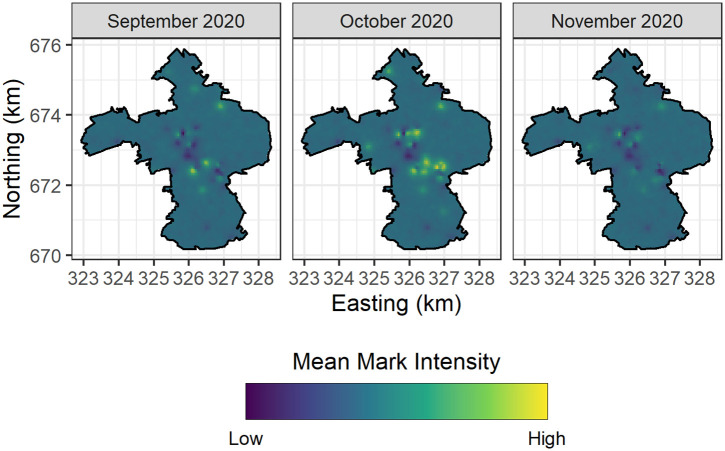
Estimated mark random field *ω*(*s*, *t*) for October-November 2020 from Model 4. Colour scale is given in low-high intensity as we are interested in relative differences across space and not absolute values.

The value for *R*_*spatiotemporal*_ is bounded between -1 and 1, where -1 suggests a strong negative correlation, 1 suggests a strong positive correlation, and 0 suggests no correlation. The posterior estimate for *R*_*spatiotemporal*_ in the chosen model suggests a positive correlation in COVID-19 levels in halls of residences over time. This means that the level of COVID-19 in a halls of residence in a given month is impacted by the COVID-19 level in that spatial area during the previous month. Because the parameter comes from a spatiotemporal field, space and time are also ‘tied together’ here, so this should be interpreted as the spatial structure of the field being correlated across time. So, if there are high levels in one area and low in another in a given month, the next month is likely to follow a similar spatial structure. Fig 5 demonstrates the correlation in the spatial structure of the mark random field (*ω*(*s*, *t*)) from Model 4 over time.

The third index, *R*_*scaling*_, can be interpreted as defining the strength and direction of the interaction between density of university halls of residence (represented by the point field *ψ*) and residence COVID-19 levels. For the chosen model, *R*_*scaling*_ is estimated to have a mean value of 1.91, with 95% CI between 0.61 and 3.28 (Table 6). This suggests that University halls of residence in areas with higher densities of University hall of residence are likely to have higher COVID-19 levels than areas with lower densities of halls.

#### Exploratory analyses

Our exploratory analyses indicate that residences for which there were relatively more fines issued also reported higher COVID-19 positivity (Spearman’s correlation coefficient = 0.34). Similarly, residences with non-ensuite-type accommodation reported higher COVID-19 positivity (Spearman’s correlation coefficient = 0.16). For the communities associated with each residence, it was noted that the Intermediate Zones with the highest positivity were associated with student residences that experienced relatively high COVID-19 positivity (Spearman’s correlation coefficient = 0.27).

#### Prior sensitivity

In order to assess the sensitivity of posterior estimates of the Gaussian Random Field parameters to prior specifications, a further 8 models were fitted. These were variations on Model 4 (selected as the most appropriate model using WAIC) in which the priors for the upper tail of the marginal standard deviation and lower tail of the range of the mark random field (*ω*) were altered. Details of the prior specifications used can be found in Table 7 and the outputs, in Table 8.

**Table 7:** Prior specifications for the standard deviation and range parameters of the mark Gaussian Random Field (*ω*) in the 9 variations of Model 4.

Model	Prior *ω* range	Prior *ω* SD
Model 4A	*P*(*ρ* < 0.2) = 0.01	*P*(*σ* > 1) = 0.01
Model 4B	*P*(*ρ* < 0.2) = 0.01	*P*(*σ* > 1.693147) = 0.01
Model 4C	*P*(*ρ* < 0.2) = 0.01	*P*(*σ* > 0.3068529) = 0.01
Model 4D	*P*(*ρ* < 0.4) = 0.01	*P*(*σ* > 1) = 0.01
Model 4E	*P*(*ρ* < 0.4) = 0.01	*P*(*σ* > 1.693147) = 0.01
Model 4F	*P*(*ρ* < 0.4) = 0.01	*P*(*σ* > 0.3068529) = 0.01
Model 4G	*P*(*ρ* < 0.1) = 0.01	*P*(*σ* > 1) = 0.01
Model 4H	*P*(*ρ* < 0.1) = 0.01	*P*(*σ* > 1.693147) = 0.01
Model 4I	*P*(*ρ* < 0.1) = 0.01	*P*(*σ* > 0.3068529) = 0.01

**Table 8:** Posterior parameter estimates for prior sensitivity test. Posterior mean and 95% credible intervals for: scaling parameter *R*_*scaling*_ (*κ*); spatial range (*R*_*spatial*_) and SD of the random fields (*ω* and *ψ*); temporal correlation (*R*_*spatiotemporal*_) of the AR1 process (*ρ*_*t*_); and regression coefficient (*β*_*i*_) of covariate: number of COVID-19 cases in the community per Intermediate Zone (*x*_*i*_). Model run times (seconds), Watanabe–Akaike information criterion (WAIC) scores, and log conditional predictive ordinate (log-CPO) scores are also given. All values given are rounded to 2 decimal places. Model 4F failed to run and so outputs for this model are not included in the table.

Parameter	Model 4A	Model 4B	Model 4C	Model 4D	Model 4E	Model 4G	Model 4H	Model 4I
*κ*	1.27	0.66	1.87	-2.65	-2.70	2.54	2.73	4.18
	[-0.04,2.61]	[-0.79,2.12]	[0.91,2.84]	[-3.88,-1.44]	[-4.11,-1.35]	[1.23,3.90]	[1.29,4.23]	[3.21,5.17]
*ω* Range	0.18	0.18	0.22	0.43	0.48	0.19	0.17	0.74
	[0.15,0.24]	[0.11,0.26]	[0.16,0.28]	[0.33,0.55]	[0.35,0.64]	[0.11,0.30]	[0.12,0.24]	[0.55,0.98]
*ω* SD	2.57	2.80	1.74	2.36	2.58	2.21	2.57	0.92
	[2.31,2.89]	[2.05,3.78]	[1.41,2.09]	[1.91,2.91]	[2.00,3.40]	[1.63,2.96]	[2.01,3.31]	[0.69,1.20]
*ψ* Range	0.47	0.46	0.45	0.44	0.45	0.45	0.45	0.42
	[0.38,0.59]	[0.38,0.55]	[0.36,0.57]	[0.34,0.55]	[0.34,0.58]	[0.34,0.59]	[0.36,0.56]	[0.35,0.51]
*ψ* SD	2.92	2.88	2.96	2.83	2.84	3.00	2.98	3.11
	[2.50,3.47]	[2.31,3.60]	[2.34,3.76]	[2.36,3.31]	[2.25,3.47]	[2.27,3.96]	[2.24,3.94]	[2.50,3.86]
*ρ*_*t*_	0.50	0.57	0.33	0.64	0.70	0.29	0.37	-0.33
	[0.43,0.57]	[0.43,0.69]	[0.16,0.50]	[0.53,0.74]	[0.60,0.78]	[0.11,0.47]	[0.22,0.51]	[-0.49,-0.14]
*β*_*i*_	-3.41	-1.99	-4.68	5.67	5.86	-6.51	-6.98	10.71
	[-47.38,40.56]	[-45.98,42.00]	[-48.60,39.23]	[-38.27,49.62]	[-38.12,49.84]	[-50.49,37.47]	[-50.98,37.02]	[-54.65,33.23]
Run Time	1042.48	1099.64	2527.84	1018.11	450.35	342.27	358.54	300.10
WAIC	1768.54	1794.07	1806.49	1733.38	1753.94	1989.86	1989.25	2585.56
log-CPO	2.6634555^6^	2.6583599^6^	2.6545139^6^	2.6102745^6^	2.6295451^6^	2.6937665^6^	2.6929661^6^	2.6909249^6^

The prior sensitivity tests indicate that the estimates of the parameters are not affected significantly by the additional variances used. If significant changes were observed, this would be interpreted as sensitivity of the estimates to the prior information/understanding of the variables at hand.

## Discussion

The advantages of the proposed approach are that (i) we interpret the Random fields of a Log Gaussian Cox Point Process to inform disease spread. Traditionally in epidemiological models, the estimation of the impact of covariates is of focus. In our study we focus on both the estimation of the impact of covariates, and the estimation of the hyperparameters of the random field. The hyperparameters of the random field are our proposed indices. As discussed earlier on, these can be adapted for the situation when there is no immunity, and adapted to facilitate comparison between spatial settings. (ii) we use both point and aggregate data—hence our marked point patterns as the data. Typically, in spatial epidemiology (reference) aggregated data is used and incidence rates are estimated (estimates) using the BYM models for example.

Recall the research questions (i) Are there any spatial patterns of COVID-19 positivity in the halls of Edinburgh University and the surrounding communities? (ii) Are there any differences in ensuite/non-ensuite positivity levels (Edinburgh University), and separately, in halls which had relatively higher/lower fines issued? (iii) What are the temporal positivity trends (in the presence of interventions such as mandatory face covering use, limitations in size of gatherings, and closure of hospitality venues) in the surrounding communities? (iv) What is the relationship between the COVID-19 positivity at halls of Edinburgh University and the community, and (v) What are the public health implications of the research findings?

We will address each research question in turn and relate the corresponding findings to public health implications.

### Research question 1

We use the terms *R*_*spatial*_ and *R*_*spatiotemporal*_ to represent the estimate of the mark field (*ω*) range and the temporal correlation (*ρ*_*t*_) respectively. These are the two of the indices that we propose in this study to describe epidemiological spread. We will now discuss the practical interpretation of these estimates in relation to COVID-19 (and in general, infectious disease) spread.

#### *R*_*spatial*_

The estimation of the range of the spatial field will give an indication of the distance over which cases are correlated with each other and serve as an estimation of the extent of the spread of the virus within halls of residence (a spatial R number). In the case of Model 4, the posterior parameter estimate is 0.19 [0.13,0.27]. This indicates that the distance over which the COVID-19 counts are spatially correlated (or related to each other) is 0.19km and the associated credible interval is 0.13km to 0.27km.

#### *R*_*spatiotemporal*_

In general, this index would give an indication of how correlated the spatial distribution of the disease under consideration is over time. Positive values for example, indicate positive correlation. The posterior estimate of the spatiotemporal correlation is 0.48 [0.37,0.59]. This indicates the correlation in the spatial distribution of COVID-19 positivity as the timeline progresses. The positive estimate of this index indicates that as the months progress from September towards December the COVID-19 positivity will be correlated to the previous month.

In terms of the spatiotemporal correlation, the practical application is that the model estimate gives us an indication of how correlated the COVID-19 positivity in one month is to that of another. In this study, we only considered 3 months. This parameter will be more important where there are more timepoints—such as 12 months where mass student vaccination took place within this timeframe. We hope to address this in a future study.

#### *R*_*scaling*_

This index defines the strength and direction of the interaction between density of university halls of residence and residence COVID-19 levels. For the chosen model, *R*_*scaling*_ is estimated to have a posterior mean value of 1.91, with 95% CI between 0.61 and 3.28. This suggests that areas with higher densities of student halls are likely to have higher COVID-19 levels than areas with lower densities of halls. This index can be useful in comparative studies where the numerical value of *R*_*scaling*_ for different universities for example, can be compared and inform related policy.

### Research questions 2 and 4

#### Exploratory analyses

Our exploratory analyses indicate that residences for which there were relatively more fines issues also reported higher COVID-19 positivity (Spearman’s correlation coefficient = 0.34). Residences with non-ensuite-type accommodation reported higher COVID-19 positivity (Spearman’s correlation coefficient = 0.16), and for the communities associated with each residence, it was noted that the Intermediate Zones with the highest positivity were associated with student residences that experienced relatively high COVID-19 positivity (Spearman’s correlation coefficient = 0.27). These are all exploratory and a larger dataset would be needed to explore these preliminary findings. The practical significance of some of these observations should not be ignored however. For example, accommodations with shared facilities (unlike ensuite residences) would be likely to facilitate more interaction between students and hence increase the probability of them being exposed to infectious disease such as COVID-19.

The abovementioned observations are important for policy and highlight the potential importance of social gatherings and the role of non-ensuite rooms in the dynamics of infectious disease spread.

#### Covariates

Four covariates were considered in this study: impact of the community, the number of fines associated with each residence, the type of accommodation (ensuite vs. non-ensuite), and whether or not the residences were part of one of the largest halls (unnamed) on campus. A statistically non-significant model estimate was obtained for each covariate. What does this suggest to policy? The statistically non-significant estimates cannot be relied upon for informing policy regarding the spread of COVID-19 amongst university halls of residence. In a future study, we plan to conduct a larger scale study which involves multiple universities in the City of Edinburgh over a longer time period. The inclusion of more data will allow us to investigate these relationships and epiaction even further.

### Research question 3

On inspection of the temporal trends and heatmaps we note that various Intermediate Zones exhibit high levels of COVID-19 positivity and in some cases, the timing of the positivity peak varies between Intermediate Zones. In this study the Dalry and Fountainbridge Intermediate Zone COVID-19 positivity appears to peak earliest, and this raises questions as to the cause of this peak. Some researchers suggest this peak is due to an outbreak at one of the universities in Edinburgh. This outbreak was followed by police enforcement of targeted policy.

Subsequent outbreaks were observed along the timeline. In most cases the outbreaks were quelled within a relatively short timeframe (in days), however the outbreak for the Intermediate Zone Old Town, Princes Street and Leith Street, during September 29th 2020 and 20th October 2020, took a relatively longer period (21 days) to decline before increasing once more. The abovementioned observations present questions to public health policy with regards to protecting communities.

The timing of the advice to students to socialize with students from their halls of accommodation coincides with the timeframe where the positivity rates are decreasing for Dalry and Fountainbridge and increasing for Marchmont East and Sciennes and Newington and Dalkeith. It is not possible to decipher the impact of the advice to students though since there are multiple dynamics at play. We can say however, that as the rates decreased in Dalry and Fountainbridge, they increased in Marchmont East and Sciennes and Newington and Dalkeith during the last two weeks of September 2020.

The fact that the effects of COVID-19 interventions are typically observed 2 weeks later suggests that it is plausible that the decreasing trends for various Intermediate Zones in early October could be due to the advice to students. The advice was for one weekend though, so it is likely that the resurgence of positivity increases in those zones could be due to resumption of mixing. The policy interventions, “a”, “b”, “c”, “d”, and “+” were all concentrated in September 2020, an indication of the dynamic response to the challenge of the rising levels of positivity.

Overall, we have shown how the use of selected hyperparameters of Log Gaussian Cox Process models can be used as epidemiological indices and can help improve the description of disease spread. In particular, we have shown how the spatial dimension can be incorporated in epidemiological indices and we have demonstrated this in a case study, alongside other important supporting analyses.

## Supporting information

S1 Fig Exploratory analyses. Exploratory analyses were used to determine trends in COVID-19 positivity against community COVID-19 levels, number of fines received by halls of residence, and proportion of ensuite rooms.
(PNG)

S1 File R Code for prior sensitivity specifications—models 1-8. (PDF)

S2 File R Code for prior sensitivity specifications—models 4A—4I. (PDF)

S1 Data Community level COVID-19 data. (ZIP)

S2 Data Shapefiles for edinburgh intermediate zones. (ZIP)
