# Supplementary figures and images for: Extending the R number by applying hyperparameters of Log Gaussian Cox process models in an epidemiological context to provide insights into COVID-19 positivity in the City of Edinburgh and in students residing at Edinburgh University
Source: PLoS One. 2023 Nov 21;18(11):e0291348. doi: 10.1371/journal.pone.0291348 (PMC10662770; doi:10.1371/journal.pone.0291348)

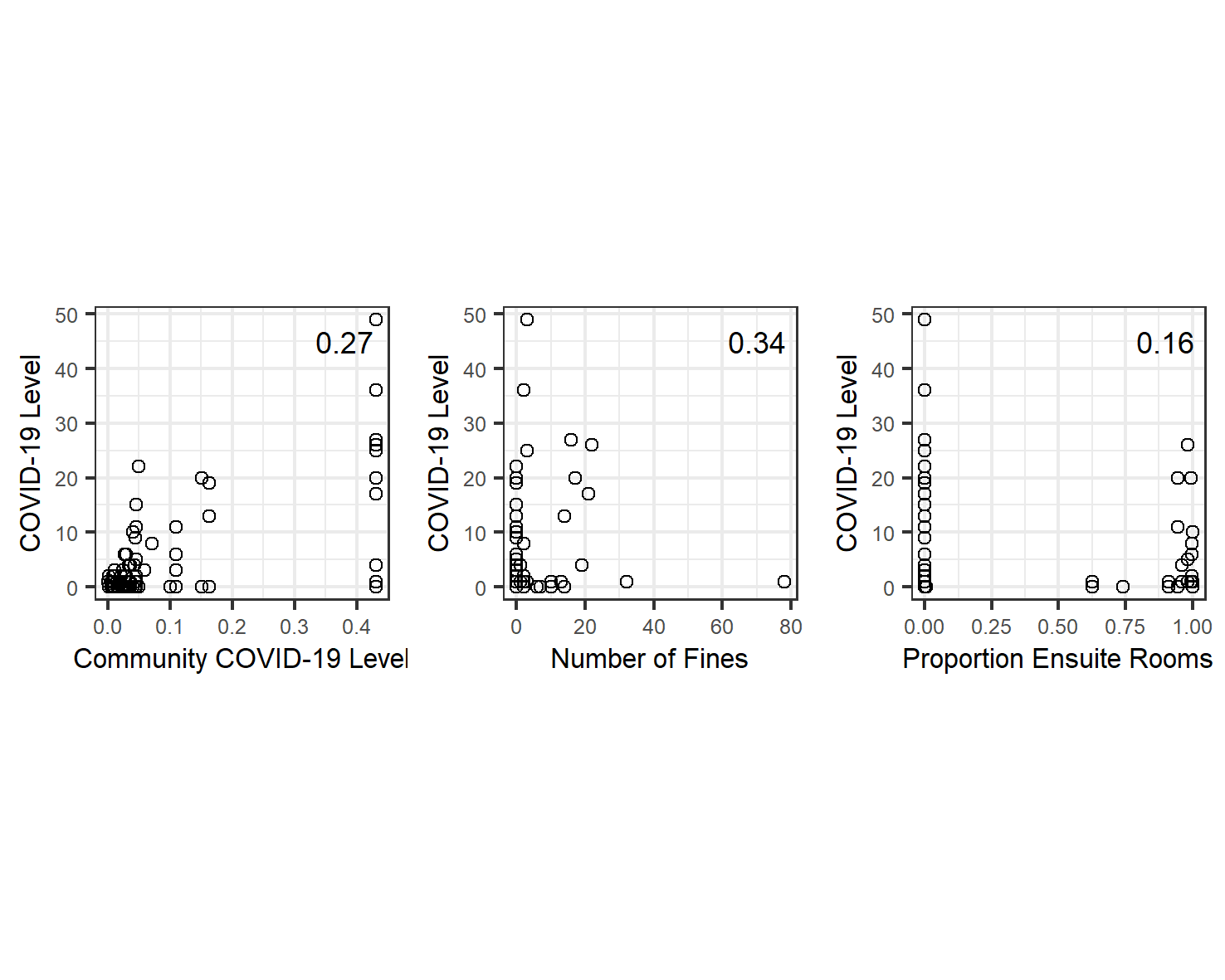

Supplement: S1 Fig — Exploratory analyses were used to determine trends in COVID-19 positivity against community COVID-19 levels, number of fines received by halls of residence, and proportion of ensuite rooms. (PNG) [file pone.0291348.s001.png]
