# Supplementary material for: Extending the R number by applying hyperparameters of Log Gaussian Cox process models in an epidemiological context to provide insights into COVID-19 positivity in the City of Edinburgh and in students residing at Edinburgh University
Source: PLoS One. 2023 Nov 21;18(11):e0291348. doi: 10.1371/journal.pone.0291348 (PMC10662770; doi:10.1371/journal.pone.0291348)
Supplement: S1 File — (PDF) [file pone.0291348.s002.pdf]

# R code - models 1-8

```r
## Set up
library(sp)
library(INLA)
library(inlabru)
library(raster)
library(dplyr)

## "pts" is a SpatialPointsDataFrame containing the locations of the
## university halls of residence and additional information about each
## hall.

## "boundary" is a SpatialPolygonsDataFrame defining the boundary of the
## area of interest.

## "comcov_pxl_spatial" is a SpatialPixelsDataFrame containing
## information on the community level of COVID-19 at each Intermediate
## Zone. "comcov_pxl" is a spatiotemporal version of this
## SpatialPixelsDataFrame, covering 3 months.

t.proj <- CRS("+proj=tmerc +lat_0=49 +lon_0=-2 +k=0.9996012717
              +x_0=400000 +y_0=-100000 +ellps=airy +units=km +no_defs")
## names target projection as British National Grid but units=km

bru_options_set(bru_verbose=3,
## info printed while model is running...
                control.compute=list(dic=TRUE,waic=TRUE,po=TRUE,cpo=TRUE))
## Compute scores for model assessment...

## Mesh
mesh <- inla.mesh.2d(boundary=boundary,
                     max.edge=c(0.1,2),
                     min.angle=c(30,21),
                     max.n=c(48000,16000),
                     max.n.strict=c(128000,128000),
                     cutoff=0.03,
                     offset=c(0.1,2),
                     crs=t.proj)

## SPDE priors
point_matern <- inla.spde2.pcmatern(mesh,
                                    prior.range=c(0.2,0.01),
                                    prior.sigma=c(1,0.01))

mark_matern <- inla.spde2.pcmatern(mesh,
                                   prior.range=c(0.2,0.01),
                                   prior.sigma=c(1,0.01))

## Define function for spatiotemporal community covariate
```

```r
f.comcov <- function(x,y,ti) {
  spp <- SpatialPoints(data.frame(x=x,y=y),
                       proj4string=fm_sp_get_crs(comcov_pxl))
  proj4string(spp) <- fm_sp_get_crs(comcov_pxl)
  v <- over(spp,comcov_pxl[which(comcov_pxl$ti==ti),])
  if (any(is.na(v$count_per_pop))) {
    v$count_per_pop <- bru_fill_missing(count_per_pop,spp,v$count_per_pop)
  }
  return(v$count_per_pop)
}

## Model 1
cmp_1 <-
  ~ -1 +
  point_field(coordinates,model=point_matern) +
  mark_field(coordinates,model=mark_matern) +
  Inter_point(1) + Inter_mark(1) +
  scaling_latent()
point_lik_1 <- like("cp",
                    formula=coordinates ~ point_field + Inter_point,
                    include=c("point_field","Inter_point"),
                    data=pts,
                    domain=list(coordinates=mesh),
                    samplers=boundary)
mark_lik_1 <- like("poisson",
                   formula=count ~ Inter_mark +
                   point_field*scaling_latent + mark_field,
                   include=c("Inter_mark","point_field","mark_field",
                             "scaling_latent"),
                   data=pts)
fit_1 <- bru(cmp_1,
             point_lik_1,
             mark_lik_1,
             options=list(verbose=TRUE,E=pts$total_rooms))

## Model 2
cmp_2 <-
  ~ -1 +
  point_field(coordinates,model=point_matern) +
  mark_field(coordinates,model=mark_matern) +
  Inter_point(1) + Inter_mark(1) +
  scaling_latent() +
  count_per_pop(comcov_pxl_spatial,model="linear")
point_lik_2 <- like("cp",
                    formula=coordinates ~ point_field + Inter_point,
                    include=c("point_field","Inter_point"),
                    data=pts,
                    domain=list(coordinates=mesh),
                    samplers=boundary)
mark_lik_2 <- like("poisson",
                   formula=count ~ Inter_mark +
                   point_field*scaling_latent + mark_field +
                   count_per_pop,
                   include=c("Inter_mark","point_field","mark_field",
                             "count_per_pop","scaling_latent"),
```

```
                   data=pts)
fit_2 <- bru(cmp_2,
             point_lik_2,
             mark_lik_2,
             options=list(verbose=TRUE,E=pts$total_rooms))

## Model 3
cmp_3 <-
  ~ -1 +
  point_field(coordinates,model=point_matern) +
  mark_field(coordinates,
             group=ti,
             group_mapper=bru_mapper_index(3),
             model=mark_matern,
             control.group=list(model="ar1")) +
  Inter_point(1) + Inter_mark(1) +
  scaling_latent()
point_lik_3 <- like("cp",
                    formula=coordinates ~ point_field + Inter_point,
                    include=c("point_field","Inter_point"),
                    data=pts,
                    domain=list(coordinates=mesh),
                    samplers=boundary)
mark_lik_3 <- like("poisson",
                   formula=count ~ Inter_mark +
                   point_field*scaling_latent + mark_field,
                   include=c("Inter_mark","point_field","mark_field",
                             "scaling_latent"),
                   data=pts)
fit_3 <- bru(cmp_3,
             point_lik_3,
             mark_lik_3,
             options=list(verbose=TRUE,E=pts$total_rooms))

## Model 4
cmp_4 <-
  ~ -1 +
  point_field(coordinates,model=point_matern) +
  mark_field(coordinates,
             group=ti,
             group_mapper=bru_mapper_index(3),
             model=mark_matern_A,
             control.group=list(model="ar1")) +
  Inter_point(1) + Inter_mark(1) +
  scaling_latent() +
  count_per_pop(f.comcov(x,y,ti),model="linear")
point_lik_4 <- like("cp",
                    formula=coordinates ~ point_field + Inter_point,
                    include=c("point_field","Inter_point"),
                    data=pts,
                    domain=list(coordinates=mesh),
                    samplers=boundary)
mark_lik_4 <- like("poisson",
                   formula=count ~ Inter_mark +
                   point_field*scaling_latent + mark_field +
```

```
                   count_per_pop,
                   include=c("Inter_mark","point_field","mark_field",
                             "count_per_pop","scaling_latent"),
                   data=pts)
fit_4 <- bru(cmp_4,
             point_lik_4,
             mark_lik_4,
             options=list(verbose=TRUE,E=pts$total_rooms))

## Model 5
cmp_5 <-
  ~ -1 +
  point_field(coordinates,model=point_matern) +
  mark_field(coordinates,
             group=ti,
             group_mapper=bru_mapper_index(3),
             model=mark_matern,
             control.group=list(model="ar1")) +
  Inter_point(1) + Inter_mark(1) +
  scaling_latent() +
  count_per_pop(f.comcov(x,y,ti),model="linear") +
  prop_ensuite
point_lik_5 <- like("cp",
                    formula=coordinates ~ point_field + Inter_point,
                    include=c("point_field","Inter_point"),
                    data=pts,
                    domain=list(coordinates=mesh),
                    samplers=boundary)
mark_lik_5 <- like("poisson",
                   formula=count ~ Inter_mark +
                   point_field*scaling_latent + mark_field +
                   count_per_pop + prop_ensuite,
                   include=c("Inter_mark","point_field","mark_field",
                   "count_per_pop","prop_ensuite",
                   "scaling_latent"),
                   data=pts)
fit_5 <- bru(cmp_5,
             point_lik_5,
             mark_lik_5,
             options=list(verbose=TRUE,E=pts$total_rooms))

## Model 6
cmp_6 <-
  ~ -1 +
  point_field(coordinates,model=point_matern) +
  mark_field(coordinates,
             group=ti,
             group_mapper=bru_mapper_index(3),
             model=mark_matern,
             control.group=list(model="ar1")) +
  Inter_point(1) + Inter_mark(1) +
  scaling_latent() +
  count_per_pop(f.comcov(x,y,ti),model="linear") +
  num_fines
point_lik_6 <- like("cp",
```

```
                    formula=coordinates ~ point_field + Inter_point,
                    include=c("point_field","Inter_point"),
                    data=pts,
                    domain=list(coordinates=mesh),
                    samplers=boundary)
mark_lik_6 <- like("poisson",
                   formula=count ~ Inter_mark +
                   point_field*scaling_latent + mark_field +
                   count_per_pop + num_fines,
                   include=c("Inter_mark","point_field","mark_field",
                             "count_per_pop","num_fines",
                             "scaling_latent"),
                   data=pts)
fit_6 <- bru(cmp_6,
             point_lik_6,
             mark_lik_6,
             options=list(verbose=TRUE,E=pts$total_rooms))

## Model 7
cmp_7 <-
  ~ -1 +
  point_field(coordinates,
              model=point_matern) +
  mark_field(coordinates,
             group=ti,
             group_mapper=bru_mapper_index(3),
             model=mark_matern,
             control.group=list(model="ar1")) +
  Inter_point(1) + Inter_mark(1) +
  scaling_latent() +
  count_per_pop(f.comcov(x,y,ti),model="linear") +
  unnamed_hall
point_lik_7 <- like("cp",
                    formula=coordinates ~ point_field + Inter_point,
                    include=c("point_field","Inter_point"),
                    data=pts,
                    domain=list(coordinates=mesh),
                    samplers=boundary)
mark_lik_7 <- like("poisson",
                   formula=count ~ Inter_mark +
                   point_field*scaling_latent + mark_field +
                   count_per_pop + unnamed_hall,
                   include=c("Inter_mark","point_field","mark_field",
                             "count_per_pop","unnamed_hall",
                             "scaling_latent"),
                   data=pts)
fit_7 <- bru(cmp_7,
             point_lik_7,
             mark_lik_7,
             options=list(verbose=TRUE,E=pts$total_rooms))

## Model 8
cmp_8 <-
  ~ -1 +
  point_field(coordinates,model=point_matern) +
```

```
  mark_field(coordinates,
             group=ti,
             group_mapper=bru_mapper_index(3),
             model=mark_matern,
             control.group=list(model="ar1")) +
  Inter_point(1) + Inter_mark(1) +
  scaling_latent() +
  count_per_pop(f.comcov(x,y,ti),model="linear") +
  unnamed_hall +
  prop_ensuite
point_lik_8 <- like("cp",
                    formula=coordinates ~ point_field + Inter_point,
                    include=c("point_field","Inter_point"),
                    data=pts,
                    domain=list(coordinates=mesh),
                    samplers=boundary)
mark_lik_8 <- like("poisson",
                   formula=count ~ Inter_mark +
                   point_field*scaling_latent + mark_field +
                   count_per_pop + unnamed_hall + prop_ensuite,
                   include=c("Inter_mark","point_field","mark_field",
                             "count_per_pop","unnamed_hall",
                             "prop_ensuite","scaling_latent"),
                   data=pts)
fit_8 <- bru(cmp_8,
             point_lik_8,
             mark_lik_8,
             options=list(verbose=TRUE,E=pts$total_rooms))

## Make Predictions
mark_field_1 <- predict(fit_1,pxl,~ mark_field,n.samples=1000)
mark_field_2 <- predict(fit_2,pxl,~ mark_field,n.samples=1000)
mark_field_3 <- predict(fit_3,pxl,~ mark_field,n.samples=1000)
mark_field_4 <- predict(fit_4,pxl,~ mark_field,n.samples=1000)
mark_field_5 <- predict(fit_5,pxl,~ mark_field,n.samples=1000)
mark_field_6 <- predict(fit_6,pxl,~ mark_field,n.samples=1000)
mark_field_7 <- predict(fit_7,pxl,~ mark_field,n.samples=1000)
mark_field_8 <- predict(fit_8,pxl,~ mark_field,n.samples=1000)

## pxl is an empty SpatialPixelsDataFrame with a column for each month
```
