# Supplementary material for: Extending the R number by applying hyperparameters of Log Gaussian Cox process models in an epidemiological context to provide insights into COVID-19 positivity in the City of Edinburgh and in students residing at Edinburgh University
Source: PLoS One. 2023 Nov 21;18(11):e0291348. doi: 10.1371/journal.pone.0291348 (PMC10662770; doi:10.1371/journal.pone.0291348)
Supplement: S2 File — (PDF) [file pone.0291348.s003.pdf]

```
## Set up
library(sp)
library(INLA)
library(inlabru)
library(raster)
library(dplyr)

## Note that spatial objects "pts", "boundary", "mesh", and "comcov_pxl",
## and function for covariate "f.comcov" are as defined in the previous
## section on Models 1-8.

bru_options_set(bru_verbose=3,
## info printed while model is running...
                control.compute=list(dic=TRUE,waic=TRUE,po=TRUE,cpo=TRUE))
## compute scores for model assessment...

## SPDE priors
point_matern <- inla.spde2.pcmatern(mesh,
                                    prior.range=c(0.2,0.01),
                                    prior.sigma=c(1,0.01))

mark_matern_4A <- inla.spde2.pcmatern(mesh,
                                      prior.range=c(0.2,0.01),
                                      prior.sigma=c(1,0.01))

mark_matern_4B <- inla.spde2.pcmatern(mesh,
                                      prior.range=c(0.2,0.01),
                                      prior.sigma=c(1.693147,0.01))

mark_matern_4C <- inla.spde2.pcmatern(mesh,
                                      prior.range=c(0.2,0.01),
                                      prior.sigma=c(0.3068529,0.01))

mark_matern_4D <- inla.spde2.pcmatern(mesh,
                                      prior.range=c(0.4,0.01),
                                      prior.sigma=c(1,0.01))

mark_matern_4E <- inla.spde2.pcmatern(mesh,
                                      prior.range=c(0.4,0.01),
                                      prior.sigma=c(1.693147,0.01))

mark_matern_4F <- inla.spde2.pcmatern(mesh,
                                      prior.range=c(0.4,0.01),
                                      prior.sigma=c(0.3068529,0.01))

mark_matern_4G <- inla.spde2.pcmatern(mesh,
                                      prior.range=c(0.1,0.01),
                                      prior.sigma=c(1,0.01))

mark_matern_4H <- inla.spde2.pcmatern(mesh,
                                      prior.range=c(0.1,0.01),
```

```
                                          prior.sigma=c(1.693147,0.01))

mark_matern_4I <- inla.spde2.pcmatern(mesh,
                                          prior.range=c(0.1,0.01),
                                          prior.sigma=c(0.3068529,0.01))

## Model 4A
cmp_4A <-
  ~ -1 +
## remove intercept; there are individual intercepts for each likelihood
  point_field(coordinates,model=point_matern) +
## field capturing spatial structure in point distribution
  mark_field(coordinates,
## field capturing spatiotemporal structure in mark distribution
              group=ti,
## group by temporal index
              group_mapper=bru_mapper_index(3),
## 3 months of data
              model=mark_matern_4A,
## using SPDE priors specified above
              control.group=list(model="ar1")) +
## AR1 temporal correlation structure
  Inter_point(1) + Inter_mark(1) +
## intercepts for each likelihood
  scaling_latent() +
## scaling parameter = interaction between point field and mark response
  count_per_pop(f.comcov(x,y,ti),model="linear")
## community covariate
point_lik_4A <- like("cp",
## likelihood for point model
                       formula=coordinates ~ point_field + Inter_point,
                       include=c("point_field","Inter_point"),
                       data=pts,
                       domain=list(coordinates=mesh),
                       samplers=boundary)
mark_lik_4A <- like("poisson",
## likelihood for mark model
                      formula=count ~ Inter_mark +
                      point_field*scaling_latent + mark_field +
                      count_per_pop,
                      include=c("Inter_mark","point_field","mark_field",
                                "count_per_pop","scaling_latent"),
                      data=pts)
fit_4A <- bru(cmp_4A,
               point_lik_4A,
               mark_lik_4A,
## fit the model with both likelihoods
               options=list(verbose=TRUE,
## print info as model is running
                            E=pts$total_rooms))
## sampling effort proxied by no. of rooms in hall

## Repeat, substituting in 4B-4I for 4A.
```
